# T cell immune abnormalities in immune thrombocytopenia

**DOI:** 10.1186/s13045-014-0072-6

**Published:** 2014-10-02

**Authors:** Xuebin Ji, Liping Zhang, Jun Peng, Ming Hou

**Affiliations:** Department of Hematology, Qilu Hospital of Shandong University, 107 West Wenhua Rd, Jinan, Shandong 250012 People’s Republic of China; Jinan Stomatological Hospital, Jinan, People’s Republic of China

**Keywords:** Immune thrombocytopenia, T cell, Immune tolerance, Pathogenesis

## Abstract

Immune thrombocytopenia is an autoimmune disease with abnormal T cell immunity. Cytotoxic T cells, abnormal T regulatory cells, helper T cell imbalance, megakaryocyte maturation abnormalities and abnormal T cell anergy are involved in the pathogenesis of this condition. The loss of T cell-mediated immune tolerance to platelet auto-antigens plays a crucial role in immune thrombocytopenia. The induction of T cell tolerance is an important mechanism by which the pathogenesis and treatment of immune thrombocytopenia can be studied. Studies regarding the roles of the new inducible costimulator signal transduction pathway, the ubiquitin proteasome pathway, and the nuclear factor kappa B signal transduction pathway in the induction of T cell tolerance can help improve our understanding of immune theory and may provide a new theoretical basis for studying the pathogenesis and treatment of immune thrombocytopenia.

## Introduction

Immune thrombocytopenia (ITP), also known as idiopathic thrombocytopenic purpura, is an immune-mediated disease of adults and children that is characterized by excessive platelet destruction and decreased platelet production. At the Italian Vicenza Consensus Conference in October 2007, the experts of the ITP International Working Group decided to use the term “immune” in place of “idiopathic” to emphasize the immune-mediated mechanism of the disease.

**Figure 1:**
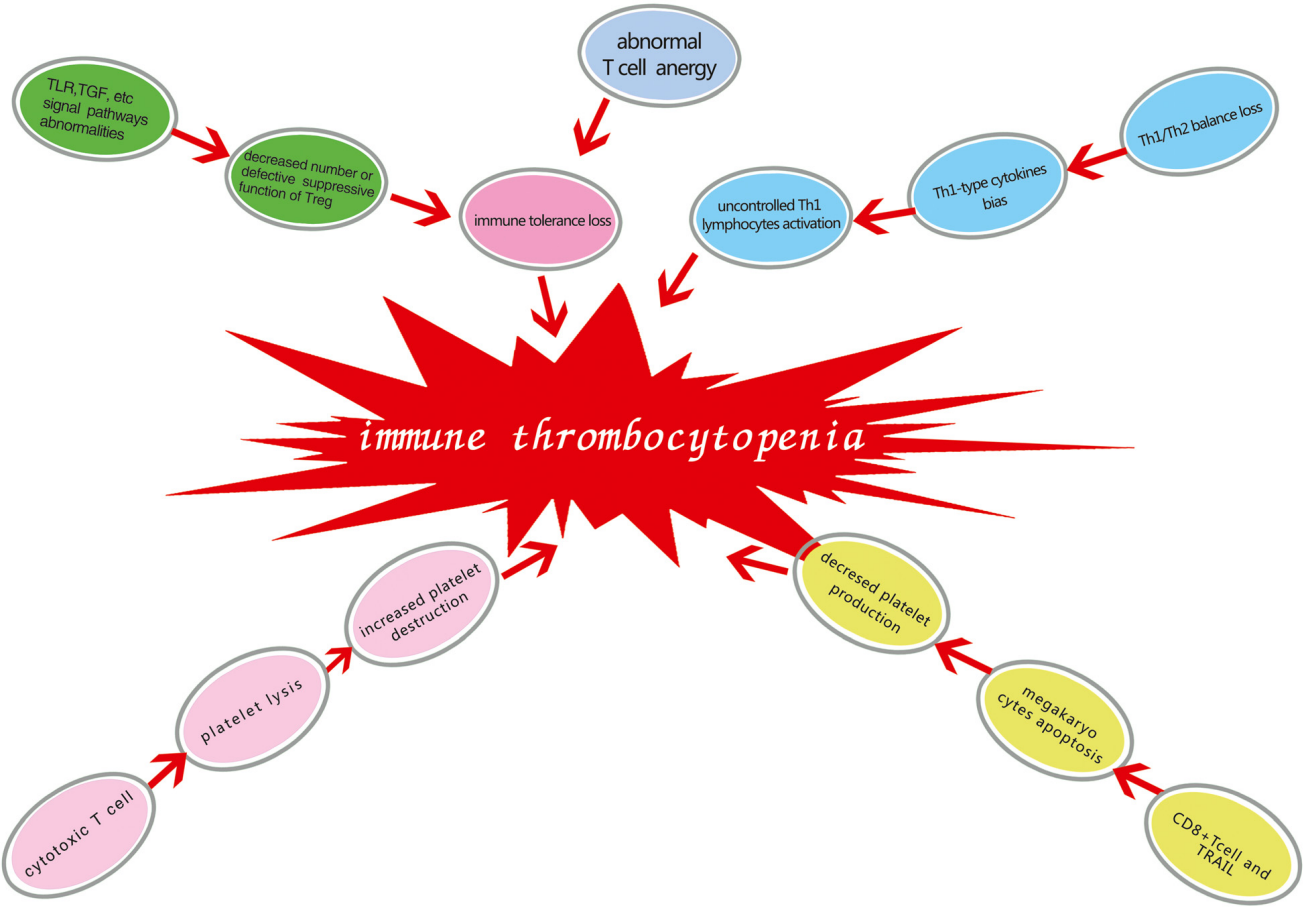
**T cell immune abnormalities in ITP pathogenesis.** Immune thrombocytopenia pathogenesis is a complicated process. T cell immune abnormalities are involved in ITP pathogenesis. These abnormalities include platelet auto-antigen reactive cytotoxic T cells, abnormal numbers and functions of T regulatory cells, loss of Th1/Th2 balance, megakaryocyte maturation abnormalities and abnormal T cell anergy.

ITP pathogenesis is very complicated. Studies of ITP pathogenesis show that the direct dissolution of antigen-specific antibodies mediates platelet destruction and that cytotoxic T cells (CTL) increase platelet destruction. In addition to platelet destruction, an immune-mediated megakaryocyte maturation abnormality and abnormal apoptosis result in decreased platelet production in ITP patients. T cell immune abnormalities play crucial roles in ITP pathogenesis. These T cell abnormalities are characterized by the excessive activation and proliferation of platelet auto-antigen-reactive CTLs [1,2], abnormal numbers and functions of T regulatory cells (Tregs) [3,4], production of abnormal helper T (Th) cells [5,6], megakaryocyte maturation abnormalities [7,8], abnormal T cell anergy [9,10], and other features [Figure 1].

ITP and thrombotic thrombocytopenic purpura (TTP) are distinct diseases that are differentially diagnosed by measuring the serum ADAMTS13 concentration. ADAMTS13 is a von Willebrand factor-cleaving protease that belongs to the ADAMTS gene family. The detection of ADAMTS-13 can provide useful information in distinguishing between ITP and TTP [11,12]. Intravenous immunoglobulin (IVIG) administration is a common treatment for ITP and TTP [13].

New studies of the roles of the ICOS/ICOS-L co-stimulatory signal transduction pathways, ubiquitin proteasome pathway and NF-κB signal transduction pathway in the induction of T cell tolerance may provide a new theoretical basis for studying the pathogenesis and treatment of ITP.

### Mechanism of action of cytotoxic T cells in ITP

CTLs play an important role in human cellular and tumor immunity. CTLs can kill tumor cells or the cells infected with bacteria or viruses by programming the cells to undergo apootosis. Memory engraftment with tumor-specific CTLs provides long-term protection against tumors [14]. Tumor cell-derived exosome-targeted dendritic cells can stimulate stronger CD8+ CTL responses and anti-tumor immunity [15]. Radioiodine gene therapy increases the cytotoxic activity of CTLs through the phenotypic modulation of tumor cells [16].

Leukotriene B4 can augment the activity of human cytotoxic cells [17]. CTL activation requires help from CD4+ T cells, which provide this help indirectly via the modification of antigen-presenting cells [18]. CD4+ T cells play an important role in the initiation and persistence of CD8+ T cell responses. Active CD4+ helper T cells directly stimulate CD8+ cytotoxic T lymphocyte responses [19].

CTLs can also induce the lysis of autologous platelets. Platelet lysis mediated by CD8+ T cells has been demonstrated to be involved in ITP pathogenesis [20,21]. In ITP patients in whom anti-platelet auto-antibodies cannot be detected, CTL-mediated platelet lysis is higher than in the healthy control group. In the remitted ITP patients, the level of platelet lysis induced by CTLs was not significantly different from that in the normal control group.

### Mechanisms of regulatory T cells in ITP

Tregs are decreased in number or can exhibit defective suppressive functions in patients with ITP. CD4 + CD25+ Tregs play critical roles in the maintenance of peripheral immune tolerance. Platelet glycoprotein (GP)-specific induced Tregs could be generated de novo from non-regulatory CD4 + CD25–CD45RA + cells in ITP patients, and these cells can induce antigen-specific immune suppression.

Tregs play crucial roles in the induction and maintenance of immune tolerance. Indirubin, a traditional Chinese medicine, has been used in the treatment of chronic myelocytic leukemia and some autoimmune diseases. The ratios of CD4 + CD25+ Treg cells or CD4 + CD25 + Foxp3+ Treg cells to CD4+ T cells in the peripheral blood, lymph nodes, and spleens of indirubin-treated mice were enhanced significantly compared with non-treated controls. Furthermore, splenic CD4 + CD25+ Treg cells from the indirubin-treated mice were able to suppress the immune response to alloantigens.

These experimental data suggest that the injection of indirubin into ITP mice significantly increases the number of peripheral blood Tregs. The number of thymus Tregs is higher in ITP mice than in healthy control mice. In addition, indirubin injection significantly increases the inhibitory function of Treg cells. These findings suggest that indirubin promotes immune tolerance in ITP [22].

The cytokine transforming growth factor-beta (TGF-β) is a central player in maintaining the immune response balance. The dominant function of TGF-β is to regulate peripheral immune homeostasis [23]. In vitro experiments using TGF-β and glycoprotein (GP) IIb/IIIa-loaded dendritic cells showed that CD4 + CD25-CD45RA + T cells could be induced to become GPIIb/IIIa-specific CD4 + CD25 + FOXP3+ regulatory T cells with immune suppressive functions. In addition, tolerogenic dendritic cells are involved in the regulation of immune functions [3].

Toll-like receptors (TLRs) play an important role in defending the host against pathogenic microbes through the induction of inflammatory cytokines and type I interferons. Furthermore, TLRs play roles in shaping pathogen-specific humoral and cellular adaptive immune responses [24].

TLRs are the major molecules involved in the immune regulatory process. TLR and TGF-signaling pathways are also involved in platelet glycoprotein-specific immune tolerance. These findings increase our understanding of the mechanisms of induction and maintenance of auto-antigen-specific immune tolerance. Antigen-specific induced Tregs for targeted immunotherapy may be a useful therapy for autoimmune diseases such as ITP [3].

### The role of the Th1/Th2 balance in ITP

The Th1/Th2 balance is important to normal human immunity. Th1 cells primarily secrete the inflammatory cytokines IL-2, IL-12, interferon-gamma (IFN-γ) and tumor necrosis factor-beta (TNF-β). These cytokines mediate immune responses associated with cytotoxicity and local inflammation and are involved in cellular immunity and the formation of delayed-type hypersensitivity inflammation. Th1 cells are also known as inflammatory T cells. Th2 cells primarily secrete the inflammatory cytokines IL-4, IL-5, IL-6 and IL-10. The main function of these cytokines is to stimulate B cell proliferation and antibody production. Th2 cells are predominantly associated with humoral immunity. Some diterpene medicines modulate the function of human dendritic cells in a manner that favors Th2 cell polarization; this modulation could have therapeutic implications for autoimmune diseases [25]. Signal transducers and activators of transcription (STAT)-6 inhibits T-bet-independent Th1 cell differentiation by suppressing IL-12-STAT-4 signaling [26]. The Notch ligand mRNA levels of human APCs are predictive of Th1/Th2-promoting activities [27].

The cause of Th1 lymphocyte bias abnormalities in ITP remains poorly understood; however, uncontrolled Th-1 lymphocyte activation may be an important mechanism of ITP. Treatment with a large dose of dexamethasone can increase the levels of IL-4, IL-10, and TGF-β. In relapsed ITP patients, the Th1-type cytokine spectrum returns to the dominant position after treatment with dexamethasone [28]. IL-18, a member of the IL-1 cytokine family, is widely expressed in monocytes/macrophages, CD4+ T cells, NK cells, and other cell types. Furthermore, IL-18 promotes Th1 responses by stimulating the production of IFN-γ by T lymphocytes and natural killer (NK) cells via IL-18R, which dominate in ITP. A previous study confirmed that the plasma and mRNA levels of both IFN-γ and IL-18 in active ITP patients were increased significantly compared with the levels in healthy controls. The balance of IL-18/IL-18BP is involved in the progression of ITP. In addition, patients with active ITP have significantly increased IL-18 levels in vivo but decreased IL-18BP levels. This result suggests that the IL-18/IL-18BP ratio is positively correlated with the Th1/Th2 ratio in ITP patients [29]. In addition, high-dose dexamethasone treatment can restore the Th1/Th2 balance by regulating the IL-18/IL-18BP ratio [30]. The expression of Notch signaling molecules and the level of IL-17 was not significantly different between the healthy controls and the ITP patients [31]. However, the number of CD3 + CD8-IL-17+ T cells was higher in ITP patients than in healthy controls. IL-21+ T cells and Th17 cells were also found to be positively correlated in ITP patients [32,33]. The expression of IL-17 was decreased in ITP patients [34,35]. In summary, ITP patients demonstrate a Th1-type cytokine bias [36].

### Immune-mediated megakaryocyte maturation abnormality

In vitro studies have provided evidence for the autoantibody-induced suppression of megakaryocytopoiesis. Megakaryocyte production and maturation are reduced in the plasma of ITP patients. ITP plasma boosts megakaryocyte quantity but impairs the quality, thereby significantly decreasing polyploidy cells and platelet release.

CD8+ T cells can significantly inhibit the apoptosis of bone marrow megakaryocytes, thereby inhibiting platelet production in vitro. The co-culture of autologous CD8+ T cells and bone marrow megakaryocytes with dexamethasone can significantly improve the inhibitory effect of CD8+ T cells on the proliferation of bone marrow megakaryocytes [7].

Tumor necrosis factor-related apoptosis-inducing ligand (TRAIL) belongs to the TNF super-family of proteins. Some studies have suggested that that TRAIL is involved in the pathogenesis of certain autoimmune diseases [37]. Decreased TRAIL levels might contribute to impaired megakaryocyte apoptosis. Human megakaryocytes cultured in vitro express TRAIL on their surfaces. Similarly, activated platelets expressed both membrane-bound and soluble TRAIL [38]. TRAIL can promote the maturation and apoptosis of megakaryocytes.

TRAIL expression on megakaryocytes and the TRAIL concentrations in the plasma and cell culture supernatants of ITP patients were decreased compared with the levels in healthy controls. Megakaryocyte apoptosis mediated by TRAIL in the plasma of ITP patients may be a potential mechanism by which the megakaryocyte number increases in vitro. These experimental results suggest that megakaryocytes in ITP exhibit a low percentage of apoptosis, a low expression of TRAIL, and a high expression of Bcl-xL. The decreased apoptosis of megakaryocytes also contributes to in vitro dysmegakaryocytopoiesis and reduced platelet production. The abnormal expression of TRAIL in plasma and TRAIL and Bcl-xL in megakaryocytes may serve important functions in the pathogenesis of impaired megakaryocyte apoptosis in ITP [8].

### Mechanisms of T cell anergy in ITP

The induction of autoreactive T cell anergy may reverse T cell-mediated abnormal immunity in ITP. Cytotoxic T lymphocyte associated antigen 4-immunoglobulin (CTLA4-Ig) and/or cyclosporine A (CsA) can induce platelet antigen-specific T cell anergy in vitro. T cell anergy can be induced by the excessive consumption of IL-2. The addition of high doses of exogenous IL-2 can reverse the anergic state of T cells. CTLA4-Ig can be used to further explore the mechanisms by which self-reactive T cells from patients with chronic ITP induce the platelet-specific anergy of T cells. Results have shown that CTLA4-Ig can induce the transformation of ITP autoreactive T cells into anergic T cells that lack immunomodulatory effects. CTLA4-Ig can further inhibit the T cell proliferation reaction of platelets. In most ITP patients, CTLA4-Ig and/or CsA induce tolerance to platelet antigens through anergy T cells. This tolerance can be overcome by stimulation with unrelated antigens, demonstrating its platelet specificity. Anergy is associated with a lack of IL-2 and can be overcome by exogenous IL-2. Hence, IL-2 suppression is crucial for the induction of platelet-specific anergy.

Therefore, inducing the anergy of autoreactive T cells may become a new therapeutic goal for the treatment of ITP. CTLA4-Ig may be used in the treatment of ITP, and combination therapy with CTLA4-Ig and CsA should be considered for treating refractory ITP [9,10].

Recent studies have shown that the loss of T cell immune tolerance is involved in the pathogenesis of ITP [39]. The recovery of T cell tolerance is an important goal in ITP treatment.

Immune tolerance refers to the unresponsiveness of the immune system after exposure and reexposure to certain antigens, while still maintaining a normal immune response to other antigens. Immune tolerance can be naturally or artificially induced. Artificial induction has important theoretical and practical significance. The current common artificial methods for the induction of tolerance include co-stimulatory signal blockage [9], oral antigen induction, antagonistic antigen induction, soluble antigen induction, and immune deviation induction. Thorough research of the ITP T cell immune tolerance pathways is crucial to the identification of new mechanisms to recover ITP tolerance. This research has important theoretical significance for the diagnosis and treatment of ITP. T cell immune tolerance may possibly be recovered through the pathways described below.

### New co-stimulatory signal pathway-inducible co-stimulator (ICOS) pathway

Blocking ICOS co-stimulation pathways to inhibit the activity of ITP T cells in immune tolerance induction provides a theoretical basis for the mechanism and treatment of ITP. Co-stimulatory signal blockage triggers T cells to produce specific immune tolerance to antigens. Previous studies have confirmed that the ITP CD28/B7 pathway is involved in T cell activation [40-42]. The B7 co-stimulation blockers CTLA4-Ig and CsA can block the co-stimulatory signal pathway and induce T cell tolerance. Blocking CD28 co-stimulation alone cannot induce T cell immune tolerance, suggesting the presence of other pathways that mediate T cell tolerance induction [2,43]. Additional research into the ICOS/ICOS-L co-stimulatory signal pathway is necessary to identify new mechanisms involved in ITP pathogenesis. Blocking the ICOS/ICOS-L co-stimulatory signal transduction pathway could be a new way to induce T cell tolerance in ITP.

ICOS is a newly discovered member of the CD28 family. ICOS-ligand (ICOS–L) interactions can enhance the T cell response to exogenous antigen, and the ICOS/ICOSL co-stimulatory signal transduction pathway serves an important function in cellular and humoral immunity [44-46]. ICOS has an important and unique function in immune responses. The CD28/B7 pathway serves important functions in the initial stage of T cell activation. In this stage,T cells are induced to produce IL-2 and promoted to proliferation. The ICOS pathway is principally involved in the differentiation and effector stages of T cell development; furthermore, this pathway regulates T cell proliferation and differentiation [45,47,48].

The ICOS/ICOS-L co-stimulatory signal pathway is important in the immune process. This pathway not only regulates the immune response of Th1 and Th2 but also promotes the proliferation and differentiation of B cells and the formation of effector cells. It can also enhance the killing effect of CTL and NK cells that is important in the secondary immune response theory.

### Inhibition of the ubiquitin proteasome pathway

Blocking the MHC class I antigen processing, presentation, and nuclear factor kappa B (NF-κB) signal transduction system can inhibit the proliferation of lymphocyte activation and induce ITP T cell tolerance. These observations may help identify new ways to achieve artificial immune tolerance induced by the proteasome system.

Proteasome inhibitors can inhibit proteasome activity and interfere with the function of cells. Particularly, they inhibit lymphocyte activation and proliferation. The proteasome is involved in the major histocompatibility complex (MHC)-I antigen presenting function by promoting lymphocyte activation [49,50] and also plays a role in NF-κB activation.

Given the functions of the proteasome in the immune system, proteasome inhibitors can alter important immune processes. First, the processing and presentation of the MHC class I antigens are blocked by proteasome inhibitor activation. As a result, the activation of lymphocytes is inhibited, and immune tolerance is induced. Second, proteasome inhibitors inhibit the proteasomal degradation of the I kappa B (IκB) protein and trigger the inactivation of NF-κB, thereby blocking the NF-κB signal transduction system, inhibiting the activation and proliferation of lymphocytes, and inducing immune tolerance.

The transcription factor NF-κB belongs to the Rel family. It is principally involved in immune and inflammatory responses, transcription, and cell proliferation and differentiation. NF-κB activation is regulated by its inhibitory factor (IκB, inhibitor of NF-κB). In the resting state, two NF-κB proteins bind a single unit of IκB to form a trimer in the cytoplasm. Extracellular stimulation causes IκB to be degraded by the proteasome ubiquitin. Then, NF-κB is dissociated from the trimer and translocates to the nucleus as a transcription factor where it regulates gene expression, cell proliferation, and differentiation [51-53].

The proteasome inhibitor bortezomib can stabilize IκB by inhibiting the proteasome 20S subunit activity, NF-κB activity, and lymphocyte proliferation and differentiation [54-58]. Therefore, the administration of bortezomib may become a new way to induce immune tolerance. This finding provides a theoretical basis for inducing ITP T cell tolerance by blocking the proteasome pathway and the NF-κB signaling pathway.

## Conclusions and future directions

ITP is an autoimmune disease with abnormal T cellular immunity. The loss of T cell tolerance plays an important role in the pathogenesis of ITP. The induction of T cell tolerance may be a promising new therapeutic strategy for the treatment of ITP. However, additional prospective studies of the roles of the ICOS/ICOS-L co-stimulatory signal transduction pathway, ubiquitin proteasome pathway, and NF-κB signal transduction pathway in the induction of T cell immune tolerance are required. Understanding these pathways may provide a new theoretical basis for the pathogenesis and treatment of ITP.
